# A High-Quality Dopant-Free Electron-Selective Passivating Contact Made from Ultra-Low Concentration Water Solution

**DOI:** 10.3390/nano12234318

**Published:** 2022-12-05

**Authors:** Linyi Zeng, Lun Cai, Zilei Wang, Nuo Chen, Zhaolang Liu, Tian Chen, Yicong Pang, Wenxian Wang, Hongwei Zhang, Qi Zhang, Zuyong Feng, Pingqi Gao

**Affiliations:** 1 School of Physics and Optoelectronic Engineering, Guangdong University of Technology, Guangzhou 510006, China; 2 Energy Technology Research Institute Co., Ltd. for Guangzhou High-Tech Zone, Guangzhou 510730, China; 3 School of Materials, Sun Yat-sen University, Shenzhen 518107, China; 4 Institute for Solar Energy Systems, School of Physics, Sun Yat-sen University, Guangzhou 510006, China; 5 Jiangsu Collaborative Innovation Center of Photovoltaic Science and Engineering, Changzhou University, Changzhou 213164, China

**Keywords:** c-Si, solar cell, electron-selective contact, dopant-free, passivation, contact resistivity

## Abstract

Crystalline silicon solar cells produced by doping processes have intrinsic shortages of high Auger recombination and/or severe parasitic optical absorption. Dopant-free carrier-selective contacts (DF-CSCs) are alternative routines for the next generation of highly efficient solar cells. However, it is difficult to achieve both good passivating and low contact resistivity for most DF-CSCs. In this paper, a high-quality dopant-free electron-selective passivating contact made from ultra-low concentration water solution is reported. Both low recombination current (*J_0_*) ~10 fA/cm^2^ and low contact resistivity (*ρ_c_*) ~31 mΩ·cm^2^ are demonstrated with this novel contact on intrinsic amorphous silicon thin film passivated n-Si. The electron selectivity is attributed to relieving of the interfacial Fermi level pinning because of dielectric properties (decaying of the metal-induced gap states (MIGS)). The full-area implementation of the novel passivating contact shows 20.4% efficiency on a prototype solar cell without an advanced lithography process. Our findings offer a very simple, cost-effective, and efficient solution for future semiconductor devices, including photovoltaics and thin-film transistors.

## 1. Introduction

Passivation contact is a key factor for high-efficiency solar cells, which facilitates majority carrier current conduction with low contact resistivity (*ρ_c_*) and promotes high open circuit voltage with low recombination current density (*J*_0_) [1]. Solar cells with intrinsic hydrogenated amorphous silicon (a-Si:H(i)) thin layer or tunnel oxide passivated contact (TOPCon) have demonstrated efficiency above 26% due to these excellent passivation contacts [2,3]. However, the traditional passivation contacts make use of highly doped regions or highly doped layers on c-Si surface, inducing other intrinsic losses such as severe Auger recombination and/or optical parasitic absorption [4,5,6]. Doping of ultra-thin (<10 nm) films is also a challenge and well-designed equipment is required [7]. Dopant-free carrier-selective contacts (DF-CSCs) give an opportunity to overcome these shortages. For example, some transition metal oxides such as WO_x_ [8], MoO_x_ [9], and VO_x_ [10] have been proven to have ultra-high work function (5–6 eV), large bandgap (~3 eV), and degenerated semiconductor properties without artificial doping. They can induce large energy band up-bending in the n-Si surface and form low contact resistivity Ohmic contact on p-Si [11,12]. In addition, these materials can be easily deposited by thermal evaporation. As for dopant-free electron-selective contacts, halides of alkaline (earth) metals and rare earth metals such as LiF_x_ [13,14], CsF_x_ [15], MgF_x_ [16], CeF_x_ [17], and GdF_x_ [18] are the most intensively investigated system. Contact resistivity as low as ~1 mΩ·cm^2^ has been reported [19]. Better performance has been achieved with the combination of thin passivation layers such as intrinsic amorphous silicon thin layer or TiO_x_ [20,21].

Solution-processed DF-CSCs are attractive due to simple equipment configuration. Poly(3,4-ethylene dioxythiophene):poly(strenesulfonate) (PEDOT:PSS) and 2,2′,7,7′-tetrakis-[*N*,*N*-di(4-methoxyphenyl)amino]-9,9′-spirobifluorene (spiro-OMeTAD) are two well-known hole-selective contacts deposited by the spin-coating method [22,23]. Thin films spin-coated from Cs_2_CO_3_ [24], PEI/PEIE [25], ZnO [26], LiAcac [27], and CaAcac [28] are electron-selective and have been explored in OLED, OPV, and c-Si. However, all the above-mentioned solutions contain some organic additives such as xylene or methanol, which are harmful to the experimenter. In addition, the organic additives themselves can also work as DF-CSCs [29,30,31] and this fact makes it more difficult to discern the mechanism behind it.

In this paper, we introduce a new dopant-free electron-selective contact made from Triton X-100 (TX-100) aqueous solution, without any toxic chemical additives. TX-100 is a commonly used non-ionic surfactant and additive often used in biochemical applications to solubilize proteins as well as in perovskite and dye-sensitized solar cells to improve performance [32,33]. The insertion of a thin film made from TX-100 between n-Si and Al turns the contact from rectifying to Ohmic and the contact resistivity (*ρ_c_*) is as low as 0.31 mΩ·cm^2^. The TX-100/Al contact is also efficient on the a-Si:H(i) passivation layer, producing both low recombination current *J*_0_ ~ 10 fA/cm^2^ and low *ρ_c_* ~ 31.45 mΩ·cm^2^. The application of this a-Si:H(i)/TX-100/Al dopant-free passivating electron-selective contact leads to an efficiency of 20.4% for the prototype solar cell. The electron selectivity of TX-100/Al is attributed to the Fermi level pinning (FLP) effect mitigated by TX-100 dielectric properties rather than the surface dipole layer formed by the TX-100 molecule.

## 2. Experimental Procedure

### 2.1. Electron-Selective Contact Fabrication

Triton X-100 (TX-100) aqueous solution (1.01 g/mL at 20 °C) was purchased from ALADDIN (Shanghai, China). No toxic organic additives, just deionized water, were used to make solutions with different concentrations from 0.50 to 20.20 mg/mL. TX-100 films were prepared by spin-coating (1000 rpm for 15 s and 4000 rpm for 60 s) and followed by a low-temperature annealed at 60 °C for 5 min in ambient air.

### 2.2. Contact Resistivity Measurement

Single-sided polished n-Si wafers, with a resistivity of 1–3 Ω·cm and a thickness of 270 μm, were used as substrates. The wafers were cleaned by the industry standard RCA clean (all used electronic-grade purity reagents are purchased from ALADDIN, Shanghai, China) and a final hydrofluoric acid (HF 5 wt%) dip for 10 s to remove native silicon oxide from the surface. After the spin-coating of TX-100 films, the Al electrodes (200 nm) were deposited by thermal evaporation in a high background vacuum (5 × 10^−4^ Pa) chamber through a shadow mask to create a TLM pattern. The contact resistivity was extracted from the voltage–current curve measured using a Keithley 2400 source meter (Keithley, OH, USA) at room temperature in darkness.

### 2.3. Passivation Measurement 

Double-sided polished n-Si wafers, with a resistivity of 1–3 Ω·cm and a thickness of 270 μm, were cleaned with RCA cleaning and HF dipping. The a-Si:H(i) layers with a thickness of 6 nm were deposited on both sides of the wafer using plasma-enhanced chemical vapor deposition (PECVD). Then the TX-100 films were spin-coated on both sides. The effective carrier lifetimes were measured by a Sinton WCT-120 photoconductance tester (Sinton, CO, USA) via quasi-steady-state photoconductance (QSSPC) methods. All lifetime values were extracted at an excess minority carrier density of 10^15^ cm^−3^.

### 2.4. Solar Cell Fabrications 

The n-type c-Si wafer with a resistivity of 1–3 Ω·cm and a thickness of 270 nm was used to fabricate the devices. Random pyramids were made by tetramethylammonium hydroxide (TMAH) and isopropyl alcohol aqueous solution at a temperature of 85 °C for 60 min. In the first stage, p-n junction solar cells were used, whose front side was doped with boron atoms at a high temperature inside a quartz tube. The p-n junction solar cells had SiN_x_ anti-reflection coating and front metallization was completed by screen printing. To achieve better passivation and higher efficiency, a-Si:H(i) passivated heterojunction solar cells were used. Layers of 6 nm a-Si:H(i) were deposited by plasma-enhanced chemical vapor deposition (PECVD). For the front, boron-doped a-Si:H(p) layers with a thickness of 10 nm were used for hole extraction, and an 80 nm ITO layer was deposited by DC sputtering. Front Ag grids with a thickness of 300 nm were fabricated by thermal evaporation.

### 2.5. Measurement and Characterization

The optical properties of the TX-100 films were measured by a UV–vis near-infrared spectrophotometer (U-4100 from Hitachi, Tokyo, Japan). The XPS measurements were performed with a Thermo Scientific Escalab 250Xi spectrometer (Thermo Fisher Scientific, Waltham, MA, USA) using the Al Kα X-ray source (hv = 1486.6 eV). The sample was cleaned using the argon cluster to remove the surface-adventitious contamination before the UPS measurement. The work function of TX-100 materials was analyzed by ultraviolet photoelectron spectroscopy (UPS) (Thermo Fisher Scientific, Waltham, MA, USA). A VS-6821M solar cell I-V tester was used to measure the current density-voltage (*J-V*) curve under simulated AM 1.5 G illumination (1000 W/m^2^) at 25 °C. The Suns-*V_oc_* results are measured with Sinton Instruments (Sinton, CO, USA). The external quantum efficiency (EQE) as well as the reflectance were carried out with the QE-R spectral test system (Enlitech, Kaohsiung, China). No voltage or light bias was applied in the quantum efficiency test.

## 3. Results and Discussion

**Figure 1:**
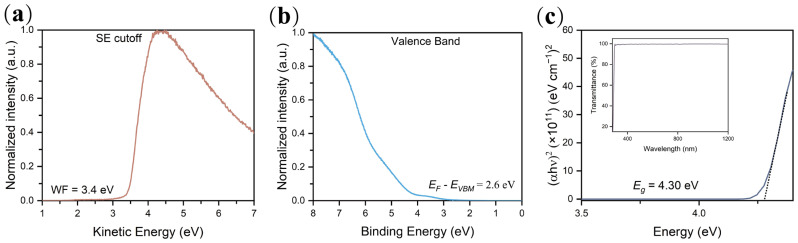
(**a**) UPS measurement results of the secondary electron cutoff spectrum and (**b**) the valence band spectrum of the TX-100 thin film; (**c**) plot of (αhν)^2^ against photon energy for the TX-100 film, the transmittance from 180 to 1200 nm is shown in the inset.

The TX-100 aqueous solution was spin-coated on a polished and slightly doped (1–3 Ω·cm) n-type c-Si wafer and then annealed at 60 °C in air for 5 min. The energy band structure of the TX-100 film was analyzed through ultraviolet photoelectron spectroscopy (UPS). The work function of the TX-100 film is 3.4 eV as indicated by the secondary electron cutoff region (Figure 1a), which is lower than the work function of Al [14]. Figure 1b shows the valence band spectrum and the valance band maximum of the TX-100 film is situated 2.6 eV below the Fermi level, which is good for blocking minority carriers. Figure 1c shows the optical transmittance and absorption spectra, which were carried out with an ultraviolet−visible (UV–vis) spectrophotometer through quartz glass covered with TX-100. The calculated optical bandgap of TX-100 is 4.3 eV, which is consistent with the literature report [34].

**Figure 2:**
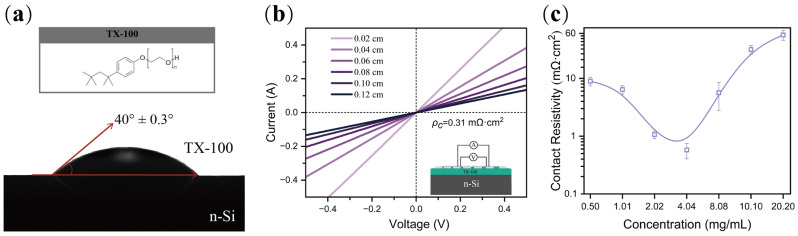
(**a**) Contact angle of TX-100 solution on HF-treated n-Si surface. The inset shows the chemical formula of TX-100; (**b**) I-V curves and calculated contact resistivity for the n-Si (1−3 Ω·cm)/TX-100 (4.04 mg/mL)/Al structure. The inset shows the TLM test structure; (**c**) relation between the *ρ_c_* value and concentration for TX-100/Al contact.

Firstly, the best concentration of the TX-100 aqueous solution was evaluated by the contact resistivity (*ρ_c_*) value by the transfer length method (TLM), which is widely used in the characterization of contact quality [35,36,37]. The contact resistivity (*ρ_c_*) values of n-Si/TX-100 (concentrations ranging from 0.50 to 20.20 mg/mL)/Al contact were extracted from the I-V curves according to TLM [38]. The existence of TX-100 thin film could be indicated by the small contact angle of water on diluted HF-treated c-Si surface, as shown in Figure 2a. The test structure of TLM is shown in Figure 2b. A champion *ρ_c_* value of 0.31 mΩ·cm^2^ is achieved, lower than most reported structures [17,18,19,39]. The contact resistance increases significantly once the optimal concentration (~4 mg/mL) deviates as shown in Figure 2c. 

**Figure 3:**
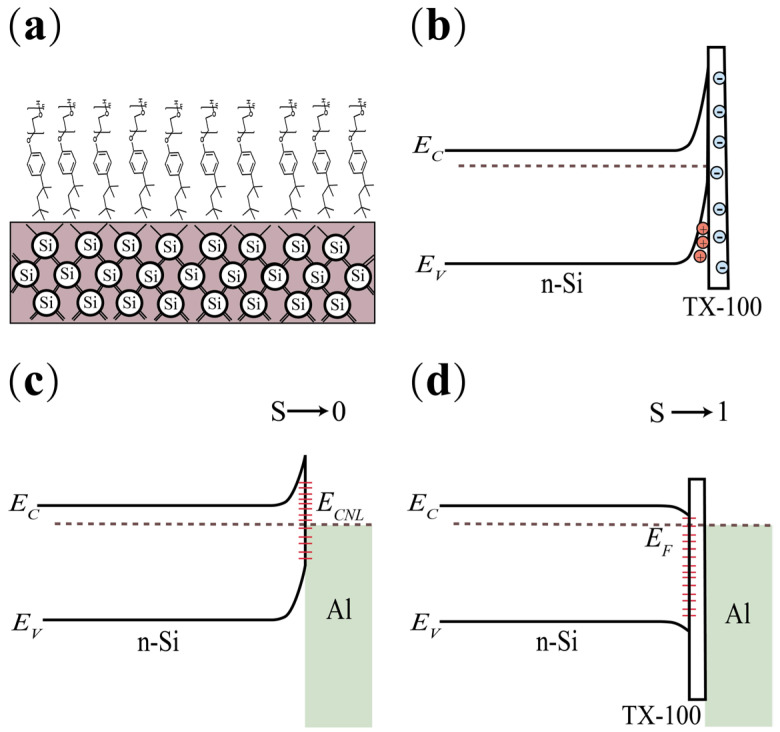
(**a**) Schematic diagram of organic molecules regularly arranged on the c-Si surface and (**b**) the resulted energy band bending for c-Si; (**c**) M-S contact, where the Fermi level of the system is pinned to a constant energy level *E_CNL_*; (**d**) the interfacial Fermi level pinning (FLP) effect mitigating by TX-100.

The improved carrier selectivity with organic solvents was often explained by surface charge accumulation, which came from ionic or strong polar groups such as amino and hydroxyl groups, fluorine-containing groups, etc. [40,41]. The schematic diagram of this statement is shown in Figure 3a. However, it is difficult to prove that most molecules are regularly arranged on the interface to form a high facial density, especially when these organic molecules are covered with thick metal electrodes (for example Al). Although TX-100 is a neutral surfactant, it does not ionize in water to produce charged ions, the silicon surface treated with HF solution is hydrophobic, thus it is reasonable to assume that the alkyl group of TX-100 stays close to the c-Si surface while the ether group tends to be far away from the surface (Figure 3a). However, as we know, the alkyl group is nearly neutral and the ether group is relatively negatively charged due to its strong electronegativity. It means the outer surface of c-Si has some negative charges. According to the principle of electrical neutrality, the energy band of c-Si should bend upward to accumulate positive charges (holes) (Figure 3b). Obviously, this inference is in contradiction with the experimental results (electron selectivity). Accordingly, we believe that ionization or spontaneously ordered charge accumulation originating from organic molecules cannot provide a convincing explanation for electron selectivity.

The Fermi level pinning (FLP) effect mitigated by dielectric materials gives a simpler and universal explanation for the results. Generally, as shown in Figure 3c, when metal contact with narrow bandgap semiconductors such as c-Si and a-Si directly, the wave function of metal penetrates the semiconductor and induces a lot of defect states, which pins the Fermi level of the system to a constant energy level *E*_CNL_. For c-Si, *E*_CNL_ is ca. 4.8 eV [42]. As a result, the actual work function of metal at the interface is *ϕ*_M,eff_.

*ϕ*_M,eff_ = S*ϕ*_M_ + (1 − S) *ϕ*_CNL_ (1)

Here, S (between 0 and 1) is the pinning factor, *ϕ*_M_ is the work function of metal measured in vacuum and *ϕ*_CNL_ is the constant energy level with respect to the vacuum. The insertion of a dielectric layer can attenuate the wave function of metal and weaken the FLP effect, as a result, S increases (closed to 1). Thus, the Schottky barrier height for the electron (*Φ*_Bn_) is:

*Φ*_Bn_ = *ϕ*_M,eff_ – χ − Δ= S*ϕ*_M_ + (1 − S) *ϕ*_CNL_ − χ − Δ (2)

where χ is the electron affinity of the semiconductor and Δ is the barrier height drop induced by image force. The barrier decreases and facilitates carrier transport. However, if the dielectric layer is too thick, then the tunneling current will decrease exponentially. As a result, the V shape curve of the contact resistivity is observed.

**Figure 4:**
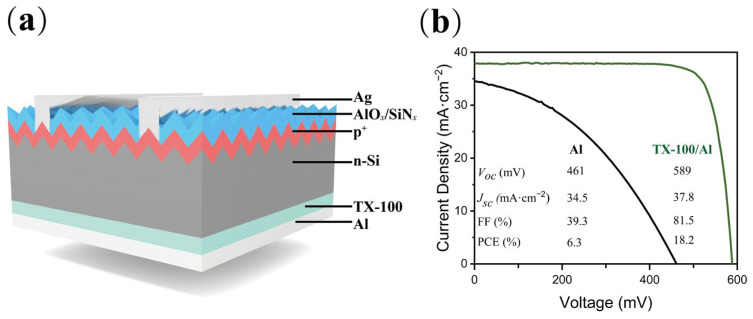
(**a**) Schematic of an n-type silicon solar cell with full-area TX-100/Al contact on the backside; (**b**) illuminated *J−V* curves without and with TX-100.

The silicon solar cells with a full-area stack of TX-100/Al rear electron contact and conventional diffused p^+^-n junction as hole contact at the front were also fabricated. The structure of the cell is shown in Figure 4a. The *J-V* curve measured at standard test condition (STC) is shown in Figure 4b. Due to the large Schottky barrier height (about 0.65 eV) [19] of n-Si/Al contact, the reference solar cell without TX-100 shows poor performance with fill factor (FF) 39.3% and power conversion efficiency (PCE or η) 6.26%. The insertion of TX-100 efficiently decreases the Schottky barrier height and promotes electron extraction, as a result, the FF increases to 81.5%, and the open circuit voltage (*V*_oc_) increases from 461 mV to 589 mV.

**Figure 5:**
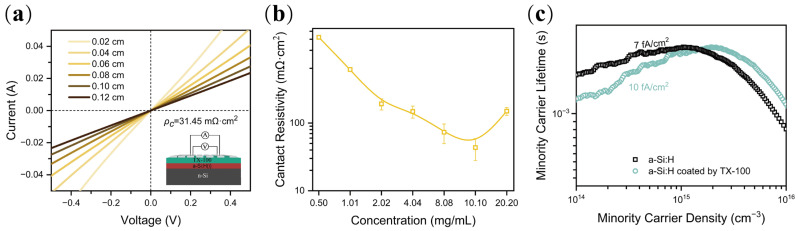
(**a**) I-V curves and calculated contact resistivity and test structure for the n-Si (1−3 Ω·cm)/a-Si:H(i)/TX-100 (10.10 mg/mL)/Al contact; (**b**) contact resistivity for different TX-100 concentration in n-Si/a-Si:H(i)/TX-100/Al contact; (**c**) passivation properties for double sides passivated n-Si wafers without and with TX-100.

Although the use of TX-100 can significantly improve solar cell performance, the efficiency is locked by the relatively large interface recombination and low *V_oc_*. To solve this problem, wafers with double sides passivated by intrinsic hydrogenated amorphous silicon (a-Si:H(i)) layers are used. Due to the poor conductivity of a-Si:H(i), the *ρ_c_* value for the n-Si/a-Si:H(i)/TX-100/Al contact is slightly increased as shown in Figure 5a, which is 31.45 mΩ·cm^2^. As shown in Figure 5b, the optimized concentration of TX-100 increased from ca. 4.04 mg/mL for n-Si/TX-100/Al contact to ca. 10.10 mg/mL for the n-Si/a-Si:H(i)/TX-100/Al hetero-contact. The results are reasonable as some potential falls in a-Si:H(i). Figure 5c shows the effective minority carrier lifetime (at the level of 10^15^ cm^−3^) of passivated silicon wafers without and with TX-100 coating. The deposit of TX-100 does not destroy the a-Si:H(i) passivation properties, as the recombination current density *J_0_* slightly increases 3 fA/cm^2^. The relatively low recombination current *J*_0_ ~10 fA/cm^2^ and low contact resistivity *ρ_c_* ~31 mΩ·cm^2^ indicate that the solar cell with a-Si:H(i)/TX-100/Al contact has the potential to achieve high efficiency.

**Figure 6:**
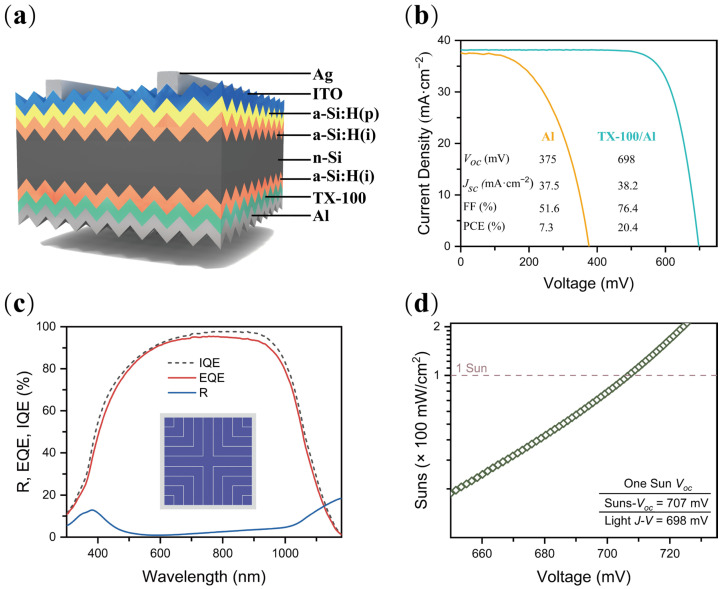
(**a**) Schematic of an n-type silicon solar cell with full-area a-Si:H(i)/TX-100/Al contact on the backside; (**b**) illuminated *J−V* curves of heterojunction solar cells without and with TX-100; (**c**) the reflectance and external/internal quantum efficiencies and (**d**) *V_oc_* of the solar cell with TX-100.

Double sides a-Si:H(i) passivated heterojunction solar cells are fabricated. The device structure is shown in Figure 6a. Figure 6b shows the *J-V* curves of heterojunction solar cells without and with TX-100. The Al directly contacted heterojunction solar cell shows poor performance as the *V_oc_*, *J_sc_*, FF, and PCE are 375 mV, 37.5 mA·cm^−2^, 51.6%, and 7.3%, respectively. The a-Si:H(i)/Al direct contact is not a good idea [14,43,44] as the metal atoms can easily diffuse through the thin a-Si:H layer and destroy the passivation [14,45]. The a-Si:H(i)/TX-100/Al contact demonstrates good performance, showing *V_oc_* close to 700 mV and the champion PCE of 20.4%, which is significant in similar investigations [25,27]. The spectrum response is shown in Figure 6c, revealing the optical design of this heterojunction solar cell could be further enhanced as the reflection (R) and internal quantum efficiency (IQE) is low in 300–600 nm. An ITO/MgF_x_ anti-reflection coating may work [46]. The illumination-dependent open circuit voltage (Suns-*V_oc_*) is shown in Figure 6d. No reverse bending has been detected in the investigated illumination region, demonstrating the Ohmic contact of a-Si:H(i)/TX-100/Al could be maintained even at high injection. The measured *V*_oc_ by Suns*V_oc_* is slightly (9 mV) higher than the one measured at STC, indicating that there is still some potential loss at the a-Si:H(i)/TX-100/Al contact. Low work function metals such as Mg and Yb may help minimize this *V*_oc_ loss.

## 4. Conclusions

In this work, a neutral surfactant, TX-100, is investigated as a high-quality dopant-free electron-selective contact for n-Si solar cells. Both low recombination current ~10 fA/cm^2^ and low contact resistivity ~31 mΩ·cm^2^ are demonstrated. Furthermore, the working principle is discussed in detail, which is attributed to Fermi level pinning relief. The heterojunction solar cell with full-area a-Si:H(i)/TX-100/Al contact exhibits a champion PCE of 20.4%. Our findings offer a very simple, cost-effective, and efficient solution for future semiconductor devices, including photovoltaics and thin-film transistors. Further work will be conducted on the environmental stability and thermal stability of the devices.

## Data Availability

The data presented in this article are available on request from the corresponding author.
